# Impact of meteorological factors on transmission of respiratory viruses across all age groups in the hot arid climate in Qatar

**DOI:** 10.3389/fpubh.2025.1568049

**Published:** 2025-06-06

**Authors:** May Husein, Salma Younes, Muthanna Samara, M. Rami Alfarra, Abdullatif Al Khal, Muna Al Maslamani, Gheyath K. Nasrallah, Einas Al Kuwari, Ali Al-kinani, Peter V. Coyle, Nader Al-Dewik

**Affiliations:** ^1^Molecular Virology Lab, Department of Laboratory Medicine and Pathology (DLMP), Hamad General Hospital (HGH), Hamad Medical Corporation (HMC), Doha, Qatar; ^2^Drug Discovery, Delivery and Patient Care Theme (DDDPC), Faculty of Science, Engineering and Computing, Kingston University London, Kingston upon Thames, United Kingdom; ^3^Department of Research, Women’s Wellness and Research Center, Hamad Medical Corporation, Doha, Qatar; ^4^Department of Psychology, Kingston University London, Kingston upon Thames, United Kingdom; ^5^Qatar Environment and Energy Research Institute, Hamad Bin Khalifa University, Doha, Qatar; ^6^Communicable Disease Center, Hamad Medical Corporation, Doha, Qatar; ^7^Biomedical Research Center, QU Health, Qatar University, Doha, Qatar; ^8^Wellcome-Wolfson Institute for Experimental Medicine, Queens University, Belfast, United Kingdom; ^9^Interim Translational Research Institute (iTRI), Hamad Medical Corporation (HMC), Doha, Qatar; ^10^Genomics and Precision Medicine (GPM), College of Health and Life Sciences (CHLS), Hamad Bin Khalifa University (HBKU), Doha, Qatar

**Keywords:** respiratory viruses, meteorological factors, epidemiology, age, gender, hot and climate

## Abstract

**Background:**

The association between meteorological parameters and viral transmission in temperate and subtropical arid climates is not fully understood. The climate in Qatar reaches extremes of heat and humidity but retains a similar pattern of transmission of respiratory viruses as in temperate climates.

**Gap statement:**

The need for a better understanding of the demographic and meteorological factors that drive the transmission of respiratory viruses in the community.

**Aim:**

To evaluate the relationship between meteorological and demographic factors on the transmission of 18 respiratory viruses in the State of Qatar.

**Materials and methods:**

In total, 355,948 nasopharyngeal swabs were tested for respiratory viruses from 31-Dec-2018 to 29-Dec-2019. The study involved 18 viruses, of which only 8 viruses were included in the analysis: ADV, hBoV, Flu-A, Flu-B, hPIV3, hMPV, HRV, and RSV. Respiratory virus prevalence was compared with local meteorological data including outdoor air temperature; dew point; wind speed; atmospheric pressure; relative humidity; solar radiation, and demographic factors, including age, gender, and nationality.

**Results:**

Transmission waves were seen for ADV, hBoV, Flu-A, Flu-B, hMPV, HRV and RSV but not with hPIV-3. Wind speed, air temperature, relative humidity, and solar radiation were significantly associated with Flu-A, Flu-B, hMPV, and RSV, which showed clear seasonality, but not with HRV, hBoV, and ADV, which had atypical seasonality and hPIV-3, which had no seasonality. Incidental associations could not be excluded and would need to be confirmed through multiple seasons. School age was the most significant demographic.

**Conclusion:**

Young children, rather than meteorological factors, served as the primary determinant of viral transmission. The proximity of 3 large viral waves to school reopening after the summer break suggested school transmission is an important contributor. The significant association of meteorological factors with viral transmission increased the risk further, reflecting the period of the year of maximum transmission. This was seen with as viruses with a clear seasonality but not with viruses with atypical or absent seasonality.

## Introduction

Respiratory virus infections often result in hospital admission and inappropriate use of antibiotics, a driver of antibiotic resistance Kabir (1). They are widely associated with acute lower respiratory tract infections (LRTIs), which constitute an important public health problem in both high and low-income countries and a leading cause of death; in 2016 alone LRTIs were linked to nearly 2.38 million deaths globally Collaborators (2). Influenza and respiratory syncytial virus (RSV) produce annual waves of transmission and are linked to over 65% of patients hospitalized with LRTIs, with significant levels of morbidity and mortality Scheltema et al. (3). The seasonal appearance of influenza and other respiratory viruses is well described in temperate climates, and seasonal transmission patterns are mostly associated with increased prevalence in winter. This is thought to be associated with increased indoor and outdoor mixing because of extremes of temperature. Children are more socially connected than adults because of schooling and are more susceptible to viral infections because of less immunity to viral infections than adults. Therefore, epidemics, within a single season or across seasons, likely involve an interplay between children and adults Bansal et al. (4). Meteorological factors may play a role indirectly by encouraging indoor mixing in hot and cold weather or directly through mucosal inflammation D’Amato et al. (5). Inflammation supports microbial adherence to the respiratory tract mucosa, impacting the respiratory microbiome and triggering infection Liu et al. (6).

Temperature also has an important effect on viral survival. The envelope of Flu-A is more stable at lower temperatures, with viability reduced at warmer temperatures Lowen et al. (7). Absolute rather than relative humidity is thought to increase Flu-A viability Pica and Bouvier (8) and Price et al. (9). Liu et al. (6) also showed a significant correlation of transmission with air temperature, atmospheric pressure, rainfall, and relative humidity, but no correlation with sunlight intensity or wind speed. In addition, the rate of lower respiratory tract infection-related hospital admissions was found to decrease with gradual increases in temperature and relative humidity. Understanding these dynamics more fully could help develop strategies to reduce their transmission.

Qatar is a city state bordering the Kingdom of Saudia Arabia with a population of 2.8 million. It is a peninsula protruding into the Persian Gulf with a flat terrain, a long coastal strip, and a significant inland desert. The climate is subtropical and arid, with low annual rainfall. The weather can be broadly grouped into three seasons: (1) warm from October to April; (2) hot from May to July; and (3) hot and humid from August to September. Temperatures range from 14°C to 22°C in the cooler months and from 32°C to 40°C in the hot months, with temperatures occasionally reaching 50C.

The aim of the present study was to examine the impact of meteorological factors on the transmission of respiratory viruses over the 12-month period of 2019. The meteorological factors studied were temperature; relative humidity; wind speed; atmospheric pressure; dew point; solar radiation. The demographic factors analyzed were gender, age and nationality.

## Materials and methods

### Study design and data collection

**Figure 1:**
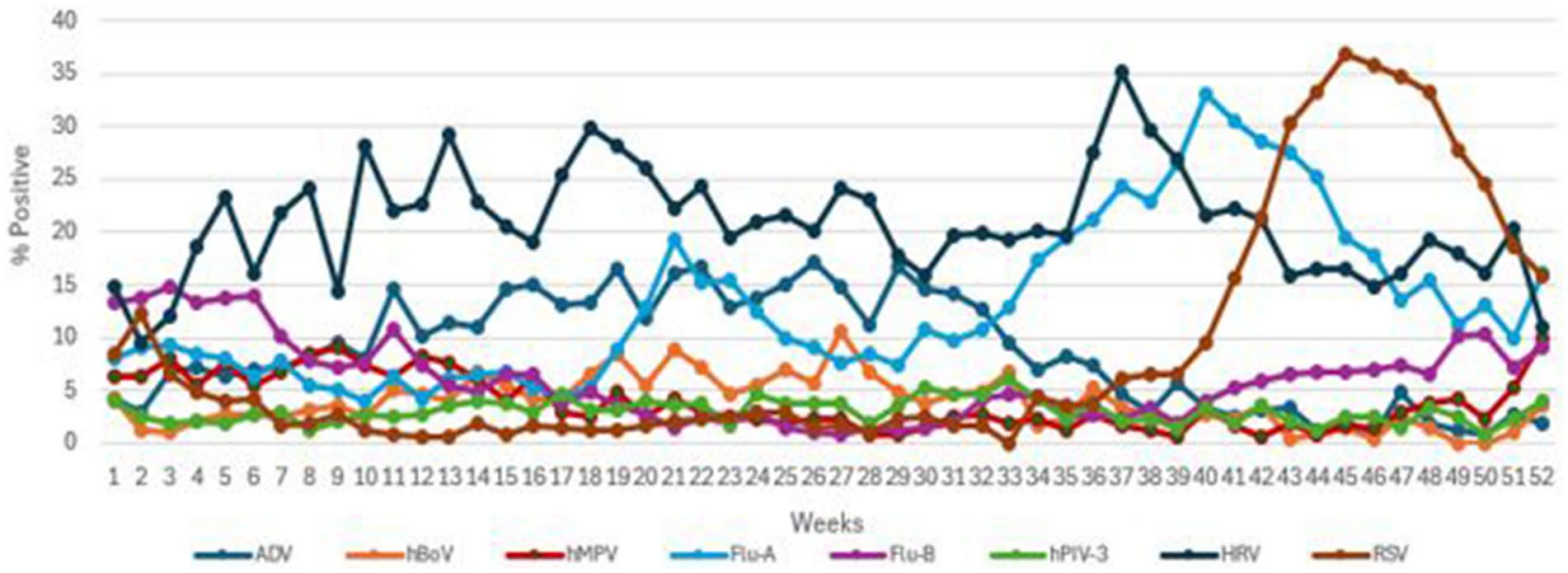
The transmission pattern of common viruses (ADV, hBoV, hMPV, Flu A, Flu B, hPIV3, HRV, and RSV) during 2019.

The results of respiratory virus testing were available for the period 1-Jan-2019 to 29-Dec-2019 and included results from 355,948 patients (Figure 1). The study population covered all age groups from multiple nationalities. Samples were tested by Real-time Polymerase Chain Reaction (qPCR) for a panel of 18 respiratory viruses and atypical bacteria using the Fast Track Diagnostics Respiratory pathogens 21 assay (***FTD™, Luxembourg***). These included: adenovirus (ADV); influenza A virus (Flu-A); influenza B virus (Flu-B); human rhinovirus (HRV); human coronaviruses (HCoV); 229E; NL63; HKU1; OC43; MERS-CoV; human parainfluenza viruses (hPIV) 1 to 4; human metapneumovirus (hMPV); human bocavirus (hBoV); respiratory syncytial virus (RSV); Parechovirus and *Mycoplasma pneumoniae* (MPN). Out of the 18 viruses, only 8 were further analyzed due to their high number of confirmed infections, based on an arbitrary threshold of 500. Samples tested were received from hospital in-patients, adult and pediatric emergency departments, primary health care centers, and outpatient clinics.

### Demographic data and meteorological factors

**Figure 2:**
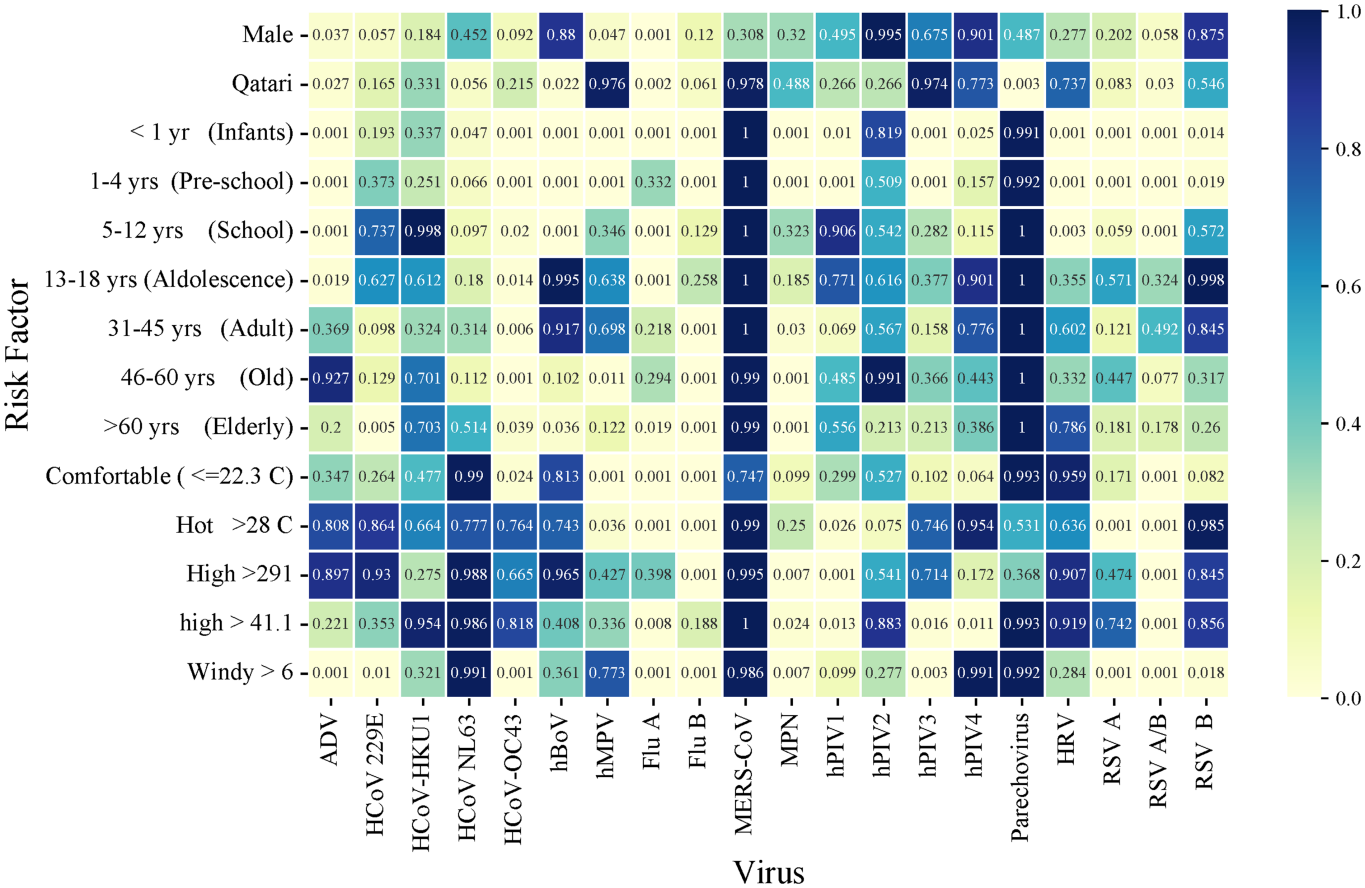
Heatmap of multivariate logistic regression analysis of virus-meteorological and virus-demographic associations. Numbers within the figure represent *p* values.

Data on average monthly outdoor meteorological factors were recorded. These included air temperature in degree Celsius (°C), dew point temperature (°C) (The temperature the air needs to be cooled in order to produce a relative humidity of 100%, which depends on the pressure and water content of the air), wind speed (Kt), atmospheric pressure (VP; hPa), % relative humidity (RH) and solar radiation (W/m^2^) (Energy emitted by the Sun, through electromagnetic waves) were obtained from the Qatar Meteorology Department weather station in Doha, situated 13 m above sea level at a Latitude of 25°16′45″N and Longitude: 51°31′20″E. The daily incidence of each pathogen by qPCR was analyzed for association with climate factors, using average monthly means of the respective meteorological parameters. Multivariate logistic regression analysis of virus, meteorological factors, and demographic are shown (Figure 2).

The study population of 355,948 patients included 150,570 females and 205,378 males divided into eight age groups respectively: < 1 year (infants); 1–4 years (pre-school age); 5–12 years (school age); 13–18 years (adolescence); 19–30 (young); 31–45 (adult); 40–60 years (old); > 60 years (older adult). Qatari nationals comprised 30.7% of the study population, and the remaining 69.3% were mainly nationals from other Arab (Egypt, Syria, Jordan, and GCC countries) and Asian (Indian, Bangladesh, and Philippines) countries, and other countries contributing smaller population sizes.

Meteorological factors were categorized based on their average monthly values throughout the year, using non-linear regression models to convert continuous variables into categorical ones through threshold-based classification. Specifically, Temperature was divided into three categories: “Comfortable” for temperatures ≤ 22.3°C, “Warm” for temperatures between 22.4°C and 27°C, and “Hot” for temperatures ≥ 28°C. Solar Radiation was classified as “High” when it exceeded 291 W/m^2^ and “Low” when it was ≤ 291 W/m^2^. Atmospheric Pressure was categorized as “High” when it was greater than 1,000 hPa and “Low” when it was ≤ 1,000 hPa. Relative Humidity was classified as “High” when it exceeded 41.1%, and “Low” when it was ≤ 41.1%. Dew Point was categorized as “Medium” when it ranged from 13.56°C to 14.82°C, “Low” when it was ≤ 13.56°C, and “High” when it was > 14.83°C. Finally, Wind Speed was classified as “Windy” when it exceeded 6 kt and “Calm” when it was ≤ 6 kt. These thresholds were established based on Qatar’s typical climate, its average weather patterns over the year, and the guidelines for wind speed classification provided by the national weather service and other relevant sources Ku-Mahamud and Khor (10). The information was gathered by the primary investigators at each location from the local Weather Spark website (weatherspark), as well as from the National Weather Service’s (weather).

### Statistical analysis

Statistical analysis used IBM SPSS 29 software (SPSS, Chicago, IL, United States). All categorical and binary variables were reported as numbers and percentages. The overall incidence of viral infections, gender, age, and meteorological factors was analyzed using Chi-Square analysis on the differences between each virus with positive and negative results. Regression analyses were performed for gender, age, nationality, and meteorological factors associated with positive results of viral infections and were reported as odds ratios (OR) with 95% confidence intervals (CI).

Univariate logistic regression was used to investigate the association of the above-mentioned factors with viral infection. Multiple logistic regression was then performed for significant variables with *p* < 0.05. Dew point and atmospheric pressure were excluded from the multiple logistic regression due to their low detection rates, which could potentially lead to redundancies with the other variables. Multiple logistic regression was then performed adjusting for all the variables. Non-significant variables were retained in the model as they interact synergistically within the same climatic system, contributing significantly to the understanding of seasonality patterns and viral dynamics. The independent risk factors included in the final model were reported as ORs with 95% CIs and statistical significance was defined as *p* < 0.05 (Table 1).

**Table 1:** Frequency differences of 17 respiratory viruses and MPN by demographic and meteorological factors over a 12-month period.

Risk factors	ADV		HCoV 229E		HCoV-HKU1		HCoV NL63		HCoV-OC43		hBoV		hMPV	
	*n* (=1843/20205)	*p*-value	*n* (=195/16940)	*p*-value	*n* (=170/16940)	*p*-value	*n* (=166/16940)	*p*-value	*n* (=326/16940)	*p*-value	n (=700/16226)	*p*-value	*n* (=643/16940)	*p*-value
Gender		0.185		0.544		0.257		0.762		0.096		**0.016**		0.081
Female (*n*)	737 (40%)		77 (39.5%)		78 (45.9%)		71 (42.8%)		121 (37.1%)		322 (46%)		289 (44.9%)	
Male (*n*)	1,106 (60%)		118 (60.5%)		92 (45.1%)		95 (57.2%)		205 (62.9%)		378 (54%)		354 (55.1%)	
Nationality		**0.031**		0.469		0.316		0.103		0.263		**0.002**		0.579
Non-Qatari (*n*)	1,259 (68.6%)		140 (72.2%)		112 (66.3%)		124 (75.6%)		236 (72.6%)		451 (64.6%)		453 (70.8%)	
Qatari (*n*)	577 (31.4%)		54 (27.8%)		57 (33.7%)		40 (24.4%)		89 (27.4%)		247 (35.4%)	0.002	187 (29.2%)	0.579
Ageband		**<0.001**		**0.034**		0.251		**<0.001**		**<0.001**		**<0.001**		**<0.001**
<1 yr. (infants)	566 (30.7%)		53 (27.2%)		53 (31.2%)		72 (43.4%)		129 (39.7%)		206 (29.4%)		198 (30.8%)	
1–4 yrs. (Pre-school)	905 (49.1%)		41 (21%)		34 (20%)		45 (27.1%)		91 (28%)		417 (59.6%)		234 (36.4%)	
5–12 yrs. (school)	196 (10.6%)		6 (3.1%)		11 (6.5%)		13 (7.8%)		17 (5.2%)		40 (5.7%)		29 (4.5%)	
13–18 yrs. (adolescence)	16 (0.9%)		3 (1.5%)		1 (0.6%)		2 (1.2%)		4 (1.2%)		2 (0.3%)		5 (0.8%)	
19–30 yrs. (Young)	46 (2.5%)		13 (6.7%)		11 (6.5%)		8 (4.8%)		13 (4.0%)		14 (2.0%)		27 (4.2%)	
31–45 yrs. (adult)	71 (3.9%)		28 (14.4%)		31 (18.2%)		13 (7.8%)		34 (10.5%)		15 (2.1%)		49 (7.6%)	
46–60 yrs. (old)	25 (1.4%)		19 (9.7%)		14 (8.2%)		8 (4.8%)		23 (7.1%)		3 (0.4%)		53 (8.2%)	
>60 yrs. (older adult)	18 (1.0%)		32 (16.4%)		15 (8.8%)		5 (3.0%)		14 (4.3%)		3 (0.4%)		48 (7.5%)	
Temperature		**<0.001**		0.171		**0.035**		**<0.001**		**<0.001**		**<0.001**		**<0.001**
Warm (22.4–27°C)	242 (13.1%)		23 (11.8%)		20 (11.8%)		13 (7.8%)		23 (7.1%)		82 (11.7%)		86 (13.4%)	
Comfortable (<=22.3°C)	506 (27.5%)		71 (36.4%)		67 (39.4%)		6 (3.6%)		190 (58.3%)		155 (22.1%)		344 (53.5%)	
Hot >28°C	1,095 (59.4%)		101 (51.8%)		83 (38.8%)		147 (88.6%)		113 (34.7%)		463 (66.1%)		213 (33.1%)	
Solar energy		**<0.001**		**0.012**		0.587		**<0.001**		0.317		**<0.001**		**<0.001**
Dark > 291	1,268 (68.8%)		133 (68.2%)		132 (77.6%)		97 (58.4%)		255 (78.2%)		479 (68.4%)		560 (87.1%)	
Bright <= 291	575 (31.2%)		62 (31.8%)		38 (22.4%)		69 (41.4%)		71 (21.8%)		221 (31.6%)		83 (12.9%)	
Pressure		**<0.001**		**0.012**		0.587		**<0.001**		0.317		**<0.001**		**<0.001**
Low <=1,000	575 (31.2%)		62 (31.8%)		38 (22.4%)		69 (41.6%)		71 (21.8%)		221 (31.6%)		83 (12.9%)	
High > 1,000	1,268 (68.8%)		133 (68.2%)		132 (77.6%)		97 (58.4%)		255 (78.2%)		479 (68.4%)		560 (87.1%)	
Humidity		**<0.001**		**0.003**		0.657		0.946		**<0.001**		**<0.001**		**<0.001**
Low <= 41.1	1,141 (61.9%)		104 (53.3%)		76 (44.7%)		71 (42.8%)		102 (31.3%)		425 (60.7%)		209 (32.5%)	
High > 41.1	702 (38.1%)		91 (46.7%)		94 (55.3%)		95 (57.2%)		224 (68.7%)		275 (39.3%)		434 (67.5%)	
Dew point		**<0.001**		**0.021**		**0.027**		**<0.001**		**<0.001**		**<0.001**		**<0.001**
Medium (13.56–14.82)	214 (11.6%)		20 (10.3%)		13 (7.6%)		1 (0.6%)		18 (5.5%)		64 (9.1%)		58 (9.0%)	
Low <= 13.56	506 (27.5%)		71 (36.4%)		67 (39.4%)		6 (3.6%)		190 (58.%)		155 (22.1%)		344 (53.5%)	
High > 14.83	1,123 (60.9%)		104 (53.3%)		90 (52.9%)		159 (95.8%)		118 (36.2%)		481 (68.7%)		241 (37.5%)	
Wind speed		**<0.001**		**<0.001**		**<0.001**		**0.049**		**<0.001**		**<0.001**		**<0.001**
Windy > 6	998 (91.5%)		112 (88.9%)		72 (72%)		28 (54.9%)		194 (87.8%)		322 (84.1%)		301 (75.3%)	
Calm <= 6	93 (8.5%)		14 (11.1%)		28 (28%)		23 (45.1%)		27 (12.2%)		61 (15.9%)		99 (24.8%)	

Risk factors	Flu-A		Flu-B		MERS-CoV		MPN		hPIV-1		hPIV-2		hPIV-3	
	*n* (=5695/40539)	*p*-value	*n* (=2741/40539)	*p*-value	*n* (=21/6073)	*p*-value	*n* (=323/16940)	*p*-value	*n* (=340/16927)	*p*-value	*n* (=84/16940)	*p*-value	*n* (=527/16940)	*p*-value
Gender		**<0.001**		0.158		**<0.001**		0.769		0.542		0.370		0.491
Female (*n*)	2,702 (47.4%)		1,168 (42.6%)		17 (81.0%)		137 (42.4%)		147 (43.2%)		39 (46.4%)		227 (43.1%)	
Male (*n*)	2,993 (52.6%)		1,573 (57.4%)		4 (19.0%)		186 (57.6%)		193 (56.8%)		45 (53.6%)		300 (56.9%)	
Nationality		**<0.001**		**0.028**		**<0.001**		0.443		0,105		**0.011**		0.451
Non-Qatari (*n*)	3,670 (64.6%)		1908 (69.8%)		5 (31.3%)		231 (71.7%)		223 (65.8%)		48 (57.1%)		360 (68.3)	
Qatari (*n*)	2012 (35.4%)		825 (30.2%)		11 (68.8%)		91 (28.3%)		116 (34.2%)		36 (42.9%)		167 (31.7%)	0.452
Ageband		**<0.001**		**<0.001**		**<0.001**		**<0.001**		**<0.001**		**0.013**		**<0.001**
<1 yr. (infants)	797 (14.0%)		384 (14.0%)		0		42 (13.0%)		122 (35.9%)		26 (31.0%)		215 (40.8%)	
1–4 yrs. (Pre-school)	2034 (35.5%)		755 (27.6%)		0		64 (19.9%)		162 (47.6%)		27 (23.1%)		193 (36.6%)	
5–12 yrs. (school)	678 (11.9%)		426 (15.5%)		5 (23.8%)		58 (18.0%)		12 (3.5%)		11 (13.1%)		13 (2.5%)	
13–18 yrs. (adolescence)	161 (2.8%)		88 (3.2%)		0		8 (2.5%)		4 (1.2%)		2 (2.4%)		3 (0.6%)	
19–30 yrs. (Young)	537 (9.4%)		561 (20.1%)		0		74 (23%)		12 (3.5%)		5 (6.0%)		28 (5.3%)	
31–45 yrs. (adult)	764 (13.4%)		424 (15.5%)		5 (23.8%)		51 (15.8%)		9 (2.6%)		6 (7.1%)		15 (2.8%)	
46–60 yrs. (old)	400 (7.0%)		61 (2.2%)		4 (19.0.%)		18 (5.6%)		9 (2.6%)		5 (6.0%)		27 (5.1%)	
>60 yrs. (older adult)	321 (5.6%)		41 (1.5%)		7 (33.3%)		7 (2.2%)		10 (2.9%)		2 (2.4%)		33 (6.3%)	
Temperature		**<0.001**		**<0.001**		0.320		0.900		**<0.001**		0.985		0.152
Warm (22.4–27°C)	926 (16.3%)		437 (15.9%)		5 (23.8%)		54 (16.7%)		42 (12.4%)		11 (13.1%)		73 (13.9%)	
Comfortable (<=22.3°C)	1,327 (23.3%)		1,671 (61.0%)		11 (52.4%)		106 (32.8%)		51 (15.0%)		26 (31.0%)		140 (26.6%)	
Hot >28°C	3,442 (60.4%)		633 (23.1%)		5 (23.4%)		163 (50.5%)		247 (72.6%)		47 (56.0%)		314 (59.6%)	
Solar energy		**<0.001**		**<0.001**		0.223		**0.006**				0.485		**0.003**
Dark > 291	4,970 (87.3%)		2,596 (94.7%)		16 (76.2%)		266 (82.4%)		256 (75.3%)		61 (72.6%)		371 (70.4%)	
Bright <= 291	725 (12.7%)		145 (5.3%)		5 (23.8%)		57 (17.6%)		84 (24.7%)		23 (27.4%)		156 (29.6%)	
Pressure		**<0.001**		**<0.001**		0.894		**0.006**		0.807		0.485		**0.003**
Low <=1,000	725 (12.7%)		145 (5.3%)		5 (23.8%)		57 (17.6%)		84 (24.7%)		23 (27.4%)		156 (29.6%)	
High > 1,000	4,970 (87.3%)		2,596 (94.7%)		16 (76.2%)		266 (82.4%)		256 (75.3%)		61 (72.6%)		371 (70.4%)	
Humidity		**<0.001**		**<0.001**		0.223		**<0.001**		**0.008**		0.360		0.000
Low <= 41.1	1,263 (22.2%)		348 (12.7%)		5 (23.8%)		177 (54.8%)		170 (50.0%)		32 (38.1%)		270 (51.2%)	
High > 41.1	4,432 (77.8%)		2,393 (87.3%)		16 (76.2%)		146 (45.2%)		170 (50.0%)		52 (61.9%)		257 (48.8%)	
Dew point		**<0.001**		**<0.001**		0.453		**<0.001**		**<0.001**		0.441		0.072
Medium (13.56–14.82)	118 (2.1%)		106 (3.9%)		NA		47 (14.6%)		33 (9.7%)		3 (3.6%)		47 (8.9%)	
Low <= 13.56	1,327 (23.3%)		1,671 (61.0%)		11 (52.4%)		106 (32.8%)		51 (15.0%)		26 (31.0%)		140 (26.6%)	
High > 14.83	4,250 (74.6%)		964 (35.2%)		10 (47.6%)		170 (52.6%)		256 (75.3%)		55 (65.5%)		340 (64.5%)	
Wind speed		**<0.001**		**<0.001**		0.154		**<0.001**		0.602		0.182		0.354
Windy > 6	818 (26.1%)		644 (41.8%)		5 (31.3%)		157 (84.9%)		98 (65.8%)		39 (76.5%)		201 (70.3%)	
Calm <= 6	2,321 (73.9%)		895 (58.2%)		11 (68.8%)		28 (15.1%)		51 (34.2%)		12 (23.5%)		85 (29.7%)	

Risk factors	hPIV-4		Parechovirus		HRV		RSV A		RSV A/B		RSV B	
	*n* (=167/16940)	*p*-value	*n* (=64/14596)	*p*-value	*n* (=1109/3980)	*p*-value	*n* (=250/3935)	*p*-value	*n* (=4238/35860)	*p*-value	*n* (=69/3935)	*p*-value
Gender		0.305		0.836		0.375		0.274		**<0.001**		0.142
Female (*n*)	76 (45.5%)		27 (42.2%)		471 (42.5%)		117 (46.8%)		1936 (45.7%)		36 (52.2%)	
Male (*n*)	91 (54.5%)		37 (57.8%)		638 (57.5%)		133 (53.2%)		2,302 (54.3%)		33 (47.8%)	
Nationality		0.792		**<0.001**		0.393		**<0.001**		**<0.001**		**<0.001**
Non-Qatari (*n*)	115 (68.9%)		29 (45.3%)		758 (68.5%)		144 (58.8%)		2,670 (63.2%)		34 (50%)	
Qatari (*n*)	52 (31.1%)		35 (54.7%)		348 (31.5%)		101 (41.2%)		1,552 (36.8%)		34 (50%)	
Ageband		**<0.001**		**<0.001**		**<0.001**		**<0.001**		**<0.001**		**<0.001**
<1 yr. (infants)	64 (38.3%)		42 (65.6%)		458 (41.3%)		146 (58.4%)		1960 (46.2%)		33 (47.8%)	
1–4 yrs. (Pre-school)	36 (21.6%)		20 (31.3%)		280 (25.3%)		62 (24.8%)		1867 (44.1%)		20 (29%)	
5–12 yrs. (school)	17 (10.2%)		2 (3.1%)		75 (6.8%)		7 (2.8%)		151 (3.6%)		1 (1.4%)	
13–18 yrs. (adolescence)	4 (2.4%)		0		11 (1.0%)		2 (0.8%)		8 (0.2%)		0	
19–30 yrs. (Young)	9 (5.4%)		0		73 (6.6%)		4 (1.6%)		51 (1.2%)		4 (5.8%)	
31–45 yrs. (adult)	10 (6.0%)		0		96 (8.7%)		13 (5.2%)		74 (1.7%)		2 (2.9%)	
46–60 yrs. (old)	16 (9.6%)		0		49 (4.4%)		5 (2.0%)		64 (1.5%)		4 (5.8%)	
>60 yrs. (older adult)	11 (6.6%)		0		66 (6.0%)		11 (4.4%)		63 (1.5%)		5 (7.2%)	
Temperature		**<0.001**		0.170		**<0.001**		**<0.001**		**<0.001**		0.130
Warm (22.4–27°C)	32 (19.2%)		6 (9.4%)		178 (16.1%)		83 (33.2%)		1,541 (36.4%)		18 (26.1%)	
Comfortable (<=22.3°C)	83 (49.7%)		27 (42.2%)		439 (39.6%)		80 (32.0%)		1,331 (31.4%)		18 (26.1%)	
Hot >28°C	52 (31.1%)		31 (48.4%)		492 (44.4%)		87 (34.8%)		1,366 (32.2%)		33 (47.8%)	
Solar energy		**0.005**		**0.047**		0.477		**<0.001**		**<0.001**		0.152
Dark > 291	142 (85.0%)		54 (84.4%)		940 (84.8%)		243 (97.2%)		4,105 (96.9%)		63 (91.3%)	
Bright <= 291	25 (15.0%)		10 (15.6%)		169 (15.2%)		7 (2.8%)		133 (3.1%)		6 (8.7%)	
Pressure		**0.005**		**0.047**		0.477		**<0.001**		**<0.001**		0.152
Low <=1,000	25 (15.0%)		10 (15.6%)		169 (15.2%)		7 (2.8%)		133 (3.1%)		6 (8.7%)	
High > 1,000	142 (85.0%)		54 (84.4%)		940 (4.8%)		243 (97.2%)		4,105 (96.9%)		63 (91.3%)	
Humidity		0.446		0.255		0.138		**<0.001**		**<0.001**		**0.008**
Low <= 41.1	67 (40.1%)		26 (40.6%)		296 (26.7%)		12 (4.8%)		218 (5.1%)		8 (11.6%)	
High > 41.1	100 (59.9%)		38 (59.4%)		813 (73.3%)		238 (95.2%)		4,020 (94.9%)		61 (88.4%)	
Dew point		**<0.001**		**0.015**		**0.012**		**<0.001**		**<0.001**		**0.006**
Medium (13.56–14.82)	28 (16.8%)		5 (7.8%)		61 (5.5%)		2 (0.8%)		34 (0.8%)		2 (2.9%)	
Low <= 13.56	83 (49.7%)		27 (42.2%)		439 (39.6%)		80 (32.0%)		1,331 (31.4%)		18 (26.1%)	
High > 14.83	56 (33.5%)		32 (50.0%)		609 (54.9%)		168 (67.2%)		2,873 (67.8%)		49 (71.0%)	
Wind speed		**<0.001**		0.080		**0.022**		**<0.001**		**<0.001**		**<0.001**
Windy > 6	81 (82.7%)		27 (87.1%)		434 (57%)		10 (5.5%)		183 (6.4%)		10 (20.8%)	
Calm <= 6	17 (17.3%)		4 (12.9%)		328 (43%)		171 (94.5%)		2,675 (93.6%)		38 (79.3%)	

* Total count of tested MERS samples are the numbers of repeated or ordered samples usually for the same patients. Bold values indicate statistical significance at *p* < 0.05.

In addition, univariate analysis of continuous variables (mean temperature, dew point, wind speed, atmospheric pressure, relative humidity, and solar radiation) were compared between days where one or more samples were, respectively, positive or negative for a given virus using two-sample *t*-tests (Table 2). Multicollinearity was observed in meteorological factors due to moderate to strong correlations among variables.

**Table 2:** Comparison of mean meteorological variables on days with and without detection of the 8 main respiratory viruses.

Agent /number of days tested	Meteorological factors	Mean of days virus was		Difference in means	95% CI/lower	Upper	*p*-value
		Not detected	Detected				
ADV	Temperature	29.079	30.481	−1.4018	−1.7173	−1.0863	**<0.001**
	Dew point	17.7286	18.3039	−0.57527	−0.83790	−0.31263	**<0.001**
	Wind speed	6.819	7.100	−0.2808	−0.3272	−0.2343	**<0.001**
	Pressure	1008.487	1006.394	2.0938	1.7562	2.4313	**<0.001**
	Humidity (relative)	43.248	39.115	4.1327	3.6820	4.5835	**<0.001**
	Solar	248.189	270.610	−22.4217	−24.8764	−19.9670	**<0.001**
hBoV	Temperature	29.788	31.26	−1.4785	−1.9720	−0.9851	**<0.001**
	Dew point	18.34	19.086	−0.70198	−1.11665	−0.28732	**<0.001**
	wind speed	6.764	6.913	−0.1497	−0.2241	−0.0754	**<0.001**
	Pressure	1007.761	1005.896	1.8646	1.3235	2.4058	**<0.001**
	Humidity (relative)	42.98	39.099	3.8827	3.1727	4.5926	**<0.001**
	Solar	251.244	270.853	−19.6086	−23.5594	−15.6578	**<0.001**
hMPV	Temperature	29.982	26.551	3.4311	2.9194	3.9428	**<0.001**
	Dew point	18.5258	15.5676	2.95818	2.52863	3.38773	**<0.001**
	Wind speed	6.758	7.054	−0.2957	−0.3725	−0.2189	**<0.001**
	Pressure	1007.561	1010.752	−3.1905	−3.7526	−2.6283	**<0.001**
	Humidity (relative)	42.721	45.085	−2.3645	−3.1058	−1.6231	**<0.001**
	Solar	252.706	236.362	16.3445	12.2262	20.4627	**<0.001**
Flu-A	Temperature	27.936	30.016	−2.0798	−2.2565	−1.9032	**<0.001**
	Dew point	17.0386	19.2820	−2.24345	−2.39189	−2.09501	**<0.001**
	Wind speed	6.636	6.139	0.4970	0.4679	0.5262	**<0.001**
	Pressure	1010.089	1009.009	1.0797	0.8881	1.2714	**<0.001**
	Humidity (relative)	45.512	46.330	−0.8180	−1.0685	−0.5675	**<0.001**
	Solar	231.501	232.243	−0.7420	−2.2506	0.7665	0.335
Flu-B	Temperature	28.460	25.035	3.4247	3.1809	3.6685	**<0.001**
	Dew point	17.5314	14.9032	2.62821	2.42215	2.83427	**<0.001**
	wind speed	6.567	6.601	−0.0346	−0.0746	0.0055	0.091
	Pressure	1009.690	1013.345	−3.6548	−3.9181	−3.3916	**<0.001**
	Humidity (relative)	45.358	49.341	−3.9826	−4.3273	−3.6380	**<0.001**
	Solar	233.411	206.698	26.7129	24.6414	28.7844	**<0.001**
hPIV-3	Temperature	29.830	30.515	−0.6849	−1.2509	−0.1189	0.018
	Dew point	18.3997	18.8413	−0.44150	−0.91679	0.03378	0.069
	wind speed	6.769	6.784	−0.0146	−0.1004	0.0713	0.740
	Pressure	1007.710	1006.839	0.8708	0.2499	1.4916	**0.006**
	Humidity (relative)	42.848	41.631	1.2170	0.4003	2.0338	**0.003**
	Solar	251.868	258.874	−7.0059	−11.5456	−2.4662	**0.002**
HRV	Temperature	27.372	28.211	−0.8384	−1.2723	−0.4045	**<0.001**
	Dew point	16.7158	17.3702	−0.65447	−1.02629	−0.28265	**<0.001**
	Wind speed	6.677	6.725	−0.0481	−0.1302	0.0340	0.251
	Pressure	1010.592	1009.646	0.9464	0.4991	1.3938	**<0.001**
	Humidity (relative)	46.718	45.798	0.9196	0.3357	1.5034	**0.002**
	Solar	226.216	234.990	−8.7740	−12.3712	−5.1769	**<0.001**
RSV A/B	Temperature	28.489	27.154	1.3350	1.1318	1.5383	**<0.001**
	Dew point	17.4841	16.9551	0.52907	0.35742	0.70072	**<0.001**
	Wind speed	6.658	5.789	0.8689	0.8363	0.9015	**<0.001**
	Pressure	1009.463	1012.737	−3.2742	−3.4927	−3.0558	**<0.001**
	Humidity (relative)	44.975	49.005	−4.0298	−4.3154	−3.7443	**<0.001**
	Solar	237.206	197.070	40.1360	38.4570	41.8150	**<0.001**

Bold values indicate statistical significance at *p* < 0.05.

An ANOVA, followed by Tukey’s post-hoc test, was conducted to determine if there were differences in the mean temperature, dew point, atmospheric pressure, relative humidity, solar radiation, and wind speed associated with the presence of various viruses.

## Results

### Viral transmission in the study population

Viruses were arbitrarily defined as the main circulating viruses where ≥500 was detected in the 12-month period. These were Flu-A, Flu-B, RSV, ADV, HRV, hMPV, hBoV, and hPIV-3. The remaining viruses and MPN, arbitrarily defined as minor circulating viruses with <500 detected were hPIV-1, OC43-CoV, HKU-COV, hPIV-4, NL63-CoV, hPIV-2, Parechovirus and MPN; 2 cases of MERS-CoV were confirmed. Data analysis was restricted to the main virus groups.

The local Qatari population made up to 34% of those tested. The male to female ratio of the patient cohort was 1.3:1, with 19,671 (5.5%) positives for one or more viruses. A total of 355,948 NPA samples were examined during the study period. Respective transmission patterns of the 8 main viruses are shown in Figure 1, with their detection frequencies in Table 1. Flu-B and hMPV had waves straddling the beginning and end of the year. The first hMPV wave peaked in week 9 and the second in week 52. The first Flu-B wave peaked in week 3 and the second in week 49. hBoV and ADV both had extended waves of 7 months from week 6 to week 40, peaking around week 26. Larger waves were seen with Flu-A, RSV and HRV. A small Flu-A wave peaked in week 21 and the larger one in week 40. RSV peaked in Week 45. HRV showed high prevalence throughout the year with a large wave peaking in week 35. hPIV-3 showed no seasonality.

### Meteorological associations

**Figure 3:**
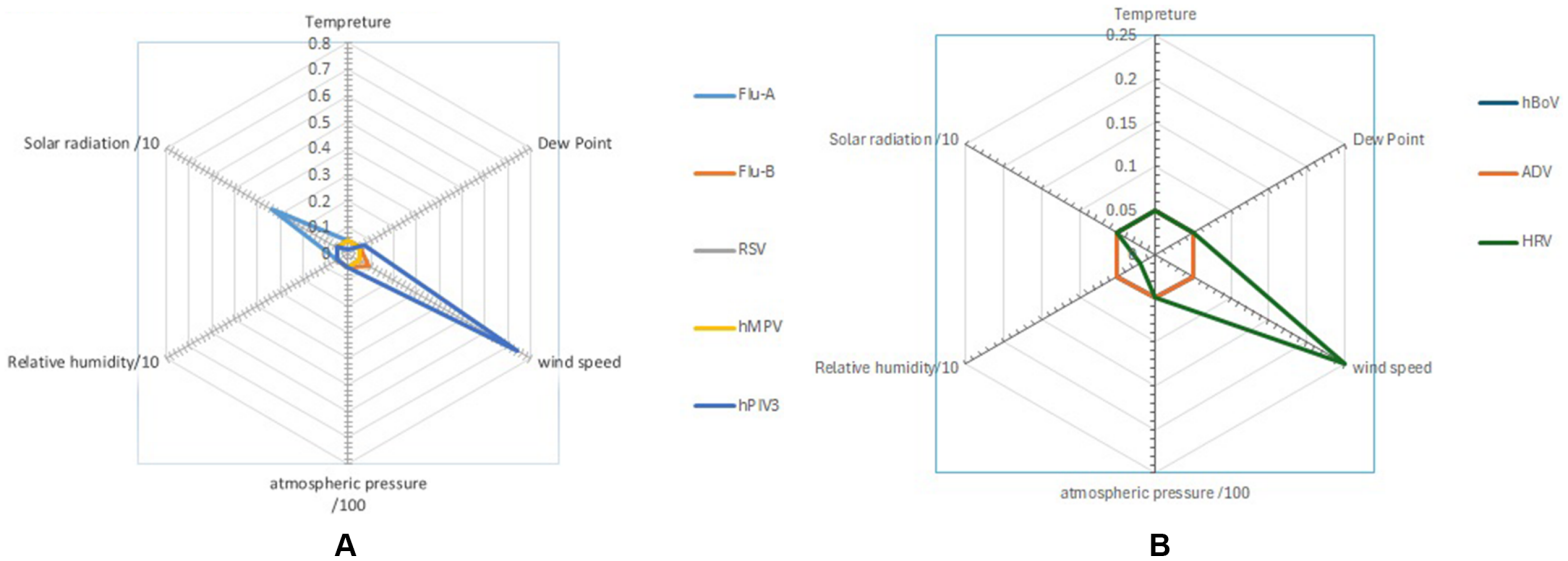
Radar chart presents the impact of dew point, relative humidity, wind speed, atmospheric pressure, and solar radiation on the main **(A)** enveloped and **(B)** non-enveloped viruses (student *T*-test).

All the main viruses showed significant correlations with all meteorological parameters except Flu-A, which did not reach significance with solar radiation and Flu-B, hPIV-3 and HRV did not reach significance with wind velocity (Table 1; Figure 3).

**Figure 4:**
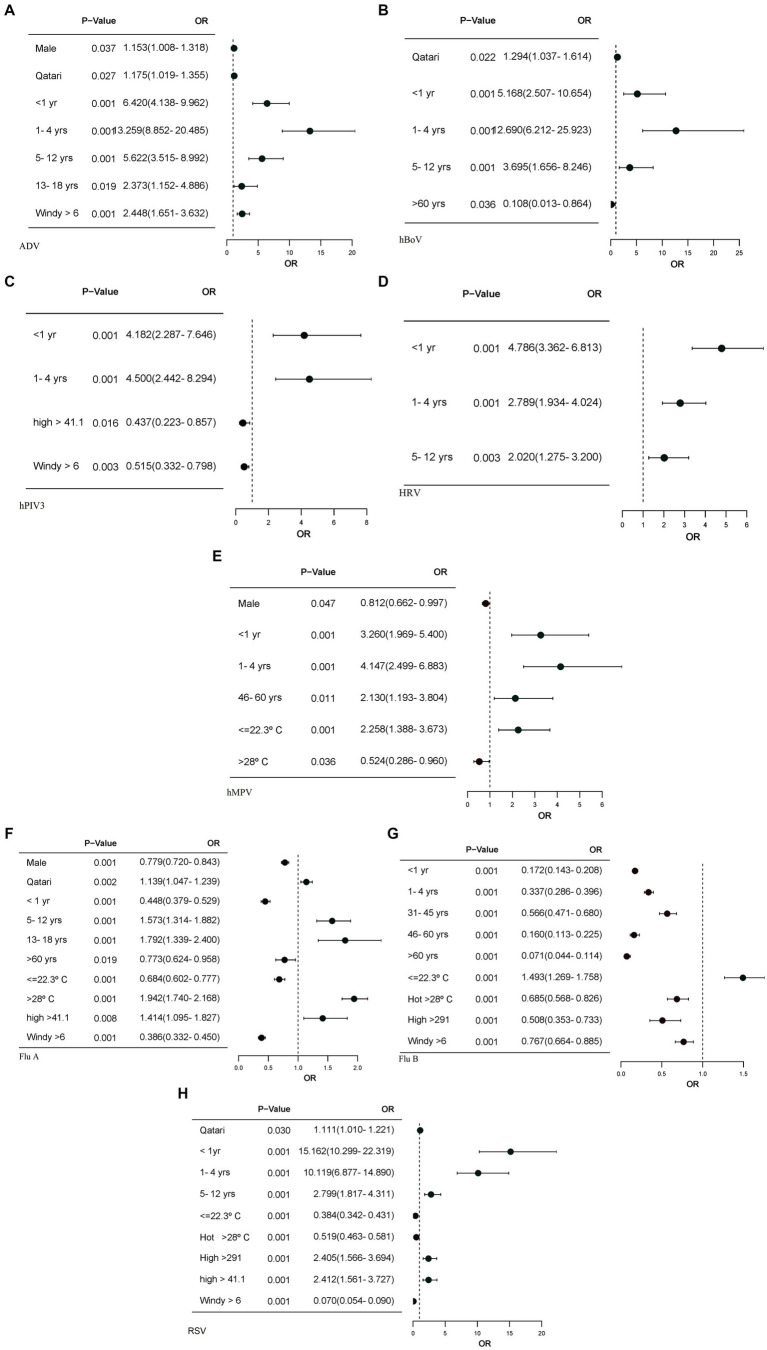
The forest plots display only the factors with significant *p*-values, along with their corresponding odds ratios. Logistic regression models were calculated for each of the meteorological, and demographic factors, adjusting for other meteorological factors. **(A)** Forest plot for ADV association with meteorological and demographic factors. **(B)** Forest plot for hBoV association with demographic factors. **(C)** Forest plot for hPIV3 association with meteorological and demographic factors. **(D)** Forest plot for HRV association with demographic factors. **(E)** Forest plot for hMPV association with meteorological and demographic factors. **(F)** Forest plot for Flu A association with meteorological and demographic factors. **(G)** Forest plot for Flu B association with meteorological and demographic factors. **(H)** Forest plot for RSV association with meteorological and demographic factors. OR, odds ratio; CI 95%, confidence interval. Windspeed >=6, RH>=50.38, solar radiation>=291, relative humidity >=41.1 and warm temperature (22.4–27°C) have been used as reference. Young age band (19–30), male gender and Qatari nationality are reference.

Wind velocity appeared to enhance the transmission of certain viruses, such as ADV, with an odds ratio (OR) of 2.448, while for other viruses, it appeared to reduce, i.e., hMPV, Flu-A, Flu-B and RSV transmitted at temperatures ≤ 22.3°C and > 28°C, suggesting that temperature did not consistently influence transmission. Relative humidity was significantly linked to Flu-A transmission (OR) of 1.414 but showed no significant association with Flu-B. Solar radiation >291 W/m^2^ was positively correlated with an increased probability of RSV transmission as reflected by an odds ratio (OR) of 2.405. In contrast, solar radiation >291 W/m^2^ was linked to a lower likelihood of Flu B transmission, with (OR) of 0.508 (Figure 4).

### Demographics

There were clear differences in the age associated with individual virus transmission. ADV, HRV and hBoV were significantly associated with transmission in newborn, pre-school, and school-age children with the highest seen, respectively, for ADV and HRV in newborns and for hBoV in pre-school children (Figures 4A,B,D). hPIV3, hMPV and Flu-A were strongly associated with infections in infants and preschool children, while RSV had the strongest association with infants. Flu-B showed negative association with age (Figures 4C,E,F,G). Male gender and Qatari nationality showed significant but differing associations with various viruses.

## Discussion

In this study, we investigated the epidemiological patterns of viral RTIs that had ≥500 confirmed infections over the 12-month period of the study in a country with a sub-tropical arid climate. Transmission was compared against demographics and meteorological data; confirmed infections acted as surrogates of viral transmission. The associations of each virus behaved as expected, providing reassurance that the observations with meteorological variables were likely to be real. The lack of seasonality of hPIV-3, in contrast to the spring/summer seasonality seen in temperate climates, deserves further study. Viruses with <500 confirmed cases were excluded.

### Demographic discussion

The large waves seen with Flu-A, RSV and HRV suggested introduction of a new variant or variants capable of triggering significant transmission. This was similar for the smaller Flu-B and hMPV waves, which straddled the respective ends of the year. The smaller summer Flu-A wave and perennial high prevalence of HRV suggested minor variant transmissions – multiple in the case of HRV. The long, 7-month, atypical waves of the non-enveloped hBoV and ADV, straddling the summer months, suggested transmission driven by a series of variants or alternative modes of transmission. These align with the findings of Chen et al. (11) who demonstrated that ADV transmission reached its peak during the summer months. In the case of ADV this could be linked to fecal-oral transmission of gastrointestinal ADVs as the qPCR assay used did not discriminate type Khanal et al. (12). The transmission pattern of hPIV3 was unique in lacking any seasonal pattern, transmitting throughout the year. This is different from transmission seen in temperate climates where it shows a spring/summer pattern, suggesting an environmental association that differs in the two climates Li et al. (13) and Xu et al. (14).

The data confirmed infants, pre-school, and school-age children were significantly associated with the transmission of all of the viruses, similarly reported by Tian et al. (15) for RSV in infants up to 6 months of age. RSV was the dominant virus in infants, and with ADV and hBoV the dominant respective virus in pre-school and school-age children. All viruses, except hPIV3, transmitted significantly in the 5–12-year age group but only ADV and Flu-A showed significant association with adolescence, aligning with the findings of Janusz et al. (16). Surprisingly, hMPV showed significant transmission in the 46–60-year age group as it is commonly regarded as sharing a similar pathophysiology to RSV. Influenza viruses showed additional significant transmission in adults and older adult patients, although individuals >60 years of age showed relatively less levels of transmission which may reflect the impact of the annual flu vaccination program. RSV, however, was not significantly seen in patients > 60 years of age, being confirmed in 1.5% of those tested. This group is now recommended to receive the new RSV vaccine because of the risk of severe disease in this age group.

Reflecting the demographic makeup of the country, there was a bias in non-Qatari persons tested, but for ADV, hBoV and RSV, transmission was significantly seen in local Qataris, reflecting the transmission pattern of these viruses in young children and the ease of access to family healthcare networks in Qatar. A similar but stronger link with Flu-A was seen and could have the same explanation and supports the use of influenza vaccine in young children. Only ADV, hMPV and Flu-A showed an association with male gender and interestingly this was not confirmed for RSV, which may contradict the received understanding that male children are more at risk of a serious clinical presentation Hall et al. (17) but reflects that most confirmed infections are not normally clinically serious.

### Meteorology discussion

Most transmission events took place in a restricted period covering the last 3 months of the year and we hypothesize that the meteorology associations during this period increased the risk of viral transmission. Temperature fluctuations for hMPV, RSV, Flu A, and Flu B were observed within the range of ≤23°C to ≥28°C. These enveloped viruses are particularly susceptible to temperature fluctuations, especially during the winter months. Polozov et al. (18) highlighted that lipid ordering in the viral envelope plays a crucial role in maintaining the virus’s stability at lower temperatures, which helps extend its survival outside the host. This lipid organization is essential for the virus to remain stable in the air, facilitating airborne transmission. Conversely, higher temperatures can disrupt the lipid membranes, impairing the virus’s ability to spread effectively Health (19). Additionally, hPIV3 has been shown to be more prevalent in conditions with lower humidity. Our observations are consistent with the findings of Price et al. (9), who noted that viruses such as ADV, Flu-A, RSV, and hMPV tend to thrive in colder environments. This understanding underscores the importance of environmental conditions, such as temperature and humidity, in influencing the transmission and survival of these viruses.

Despite the absence of a winter season in non-temperate climates, Goes et al. (20) discussed the lack of hMPV detections and the absence of typical flu seasonal patterns. However, RSV and influenza infections are primarily observed during the rainy seasons in regions of Asia, Africa, and South America Shek and Lee (21). Both hMPV and Flu-B were associated with increased transmission in cooler months, with respective ODs of 2.258 and 1.493, in keeping with transmission during the cooler months at the respective ends of the 12 months of the study. In contrast, Flu A transmission appeared to increase by warmer temperatures, with a mean temperature of 30.02°C, which could reflect the effect of the small summer wave (Table 2; Figures 3, 4).

Solar radiation was found to be associated with RSV transmission, particularly linked to the start of its transmission wave in late summer. Similarly, lower solar radiation was associated with the transmission of Flu-B (*p* < 0.001), which was explained by Flu-B primarily spreading during the winter months. This association is especially related to the seasonality of the viruses, particularly during early spring, which often happens with the shift from winter to spring season.

Relative humidity was typically higher in the later months of the year, allowing a significant correlation to be observed with the transmission of Flu A and RSV that was transmitted predominantly at this time. Elevated humidity is thought to sustain the airborne duration of aerosolized respiratory droplets, allowing them to travel further and increase the likelihood of virus transmission between individuals as seen with Flu-A (Figure 4F) and RSV (Figure 4H). This observation supported the findings of Kramer et al. (22) which reported transmission rates of both influenza and RSV are reduced by lower temperatures when absolute humidity is accounted for, and conversely are increased by higher absolute humidity when temperature is controlled. However, since most respiratory viral transmission takes place indoors over relatively short distances, increasing the length of airborne viability of viruses in respiratory droplets, coupled with a large atmospheric dilution factor, would make any association potentially incidental.

There was no consistent relationship between wind speed and transmission patterns, as some viruses showed increased transmission while others had reduced or no effect. Jiang et al. (23) proposed that strong wind speeds can carry larger droplets over greater distances, thereby increasing the risk of exposure to individuals downstream. Our findings showed that when wind speeds >6 kt, the odds ratio (OR) for ADV increased to 2.448, indicating a higher likelihood of transmission, which aligns with the results of Oh et al. (24). In contrast, the OR for other viruses is < 1.00, suggesting that higher wind speeds may either reduce or have no impact on their transmission. Xu et al. (14) found that only Coronaviruses in southern China show a relationship with wind speed. Additionally, certain viruses were identified at lower wind speeds, suggesting that outdoor transmission of COVID-19 could occur, with the risk being greater on calm days in the summer, as highlighted by Clouston et al. (25). For Flu-A, Flu-B, and RSV, it seemed that wind speeds > 6 kt reduced transmission, which aligns with findings from Zhu et al. (26). However, for HRV and hPIV3, which transmitted year-round without a seasonal pattern, no association with wind speed was observed, as shown in Figure 1. This transmission pattern of hPIV3 aligns with the findings reported by Cui et al. (27). Comparing Flu A and RSV, which had seasonal patterns, with HRV and hPIV3, which did not have seasonal patterns, revealed a stronger apparent link between the former viruses and meteorological factors, as seen in Table 2. The absence of wind speed’s impact on HRV and hPIV3 reflects their constant year-round transmission, while the apparent connection between solar energy and Flu A is probably due to an additional summer wave that occurred that year, as shown in Figure 3.

The research findings in this study can be generalized to other tropical arid climate regions that have similar climate and meteorological conditions.

## Conclusion

The results showed that school-aged children were significantly impacted by the transmission of the virus, suggesting that school attendance may have played a key role in facilitating its spread over the 12-month study period. Additionally, the observed correlation between viral transmission and meteorological factors—such as temperature, humidity, and seasonal changes—indicates that environmental conditions may have further increased the risk of transmission among this age group.

## Data Availability

The original contributions presented in the study are included in the article/supplementary material, further inquiries can be directed to the corresponding authors.

## Glossary

AQI: Air Quality Index
URTI: Upper respiratory tract infection
LRTI: Lower respiratory tract infection
RSV: Respiratory syncytial virus
RSV A: Respiratory syncytial virus A
RSV B: Respiratory syncytial virus B
COVID-19: Coronavirus disease 2019
SARS-CoV-2: Severe acute respiratory syndrome coronavirus 2
RT-PCR: Real-time polymerase chain reaction
ARI: Acute respiratory infection
Flu-A: Influenza A
Flu-B: Influenza B
ADV: Adenovirus
MPN: Mycoplasma pneumonia
MERS-CoV: Middle East Respiratory Syndrome Coronavirus
hBoV: Human bocavirus type
hCoV: Human coronavirus
HCoV 229E: *Human coronavirus 229E*
HCoV NL63: Human coronavirus NL63
HCoV-HKU1: *Human coronavirus HKU1*
HCoV-OC43: Human coronavirus OC43
hMPV: Human metapneumovirus
HPIV: Human parainfluenza virus
hPIV-1: Human parainfluenza 1
hPIV-2: Human parainfluenza 2
hPIV-3: Human parainfluenza 3
hPIV-4: Human parainfluenza 4
HRV: Human rhinovirus
ILI: Influenza-like illness
VP: Vapour Pressure
hPa: hectopascals
W/m^2^: Watts per square meter
